# ZnO Nanoparticles Induced Caspase-Dependent Apoptosis in Gingival Squamous Cell Carcinoma through Mitochondrial Dysfunction and p70S6K Signaling Pathway

**DOI:** 10.3390/ijms21051612

**Published:** 2020-02-26

**Authors:** Shih-Wei Wang, Chien-Hsing Lee, Ming-Shen Lin, Chih-Wen Chi, Yu-Jen Chen, Guo-Shou Wang, Kuang-Wen Liao, Li-Pin Chiu, Shu-Hui Wu, Dong-Ming Huang, Luke Chen, Yung-Shuen Shen

**Affiliations:** 1 Department of Medicine, MacKay Medical College, New Taipei City 252, Taiwan; shihwei@mmc.edu.tw; 2 Graduate Institute of Natural Products, College of Pharmacy, Kaohsiung Medical University, Kaohsiung 807, Taiwan; 3 Department of Pharmacology, Graduate Institute of Medicine, College of Medicine, Kaohsiung Medical University, Kaohsiung 807, Taiwan; chlee0818@kmu.edu.tw; 4 Department of Medical Research, Kaohsiung Medical University Hospital, Kaohsiung 807, Taiwan; 5 Department of Water Resources and Environmental Engineering, Tamkang University, New Taipei City 251, Taiwan; schumaher02@hotmail.com; 6 Department of Nursing, MacKay Medical College, New Taipei City 252, Taiwan; cwchid48906003@gmail.com; 7 Department of Medical Research, MacKay Memorial Hospital, Taipei 104, Taiwan; 8 MacKay Junior College of Medicine, Nursing, and Management, Taipei 112, Taiwan; chenmdphd@gmail.com; 9 Department of Radiation Oncology, MacKay Memorial Hospital, Taipei 104, Taiwan; 10 Department of Biological Science and Technology, National Chiao Tung University, Hsinchu 300, Taiwan; b8905043@gmail.com (G.-S.W.); liaonms@pchome.com.tw (K.-W.L.); 11 Department of Orthopaedics, MacKay Memorial Hospital, Taipei 104, Taiwan; 12 Institute of Molecular Medicine and Bioengineering, National Chiao Tung University, Hsinchu 300, Taiwan; 13 General Education Center, University of Taipei, Taipei 100, Taiwan; B2400@tpech.gov.tw; 14 Division of General Surgery, Taipei City Hospital, Taipei 103, Taiwan; 15 Institute of Biomedical Engineering and Nanomedicine, National Health Research Institutes, Miaoli County 350, Taiwan; shwu@nhri.edu.tw; 16 Institute of Geriatric Welfare Technology and Science, MacKay Medical College, New Taipei City 252, Taiwan

**Keywords:** zinc oxide nanoparticles, gingival cancer, superoxide, p70S6K pathway

## Abstract

Zinc oxide nanoparticles (ZnO-NPs) are increasingly used in sunscreens, food additives, pigments, rubber manufacture, and electronic materials. Several studies have shown that ZnO-NPs inhibit cell growth and induce apoptosis by the production of oxidative stress in a variety of human cancer cells. However, the anti-cancer property and molecular mechanism of ZnO-NPs in human gingival squamous cell carcinoma (GSCC) are not fully understood. In this study, we found that ZnO-NPs induced growth inhibition of GSCC (Ca9-22 and OECM-1 cells), but no damage in human normal keratinocytes (HaCaT cells) and gingival fibroblasts (HGF-1 cells). ZnO-NPs caused apoptotic cell death of GSCC in a concentration-dependent manner by the quantitative assessment of oligonucleosomal DNA fragmentation. Flow cytometric analysis of cell cycle progression revealed that sub-G1 phase accumulation was dramatically induced by ZnO-NPs. In addition, ZnO-NPs increased the intracellular reactive oxygen species and specifically superoxide levels, and also decreased the mitochondrial membrane potential. ZnO-NPs further activated apoptotic cell death via the caspase cascades. Importantly, anti-oxidant and caspase inhibitor clearly prevented ZnO-NP-induced cell death, indicating the fact that superoxide-induced mitochondrial dysfunction is associated with the ZnO-NP-mediated caspase-dependent apoptosis in human GSCC. Moreover, ZnO-NPs significantly inhibited the phosphorylation of ribosomal protein S6 kinase (p70S6K kinase). In a corollary in vivo study, our results demonstrated that ZnO-NPs possessed an anti-cancer effect in a zebrafish xenograft model. Collectively, these results suggest that ZnO-NPs induce apoptosis through the mitochondrial oxidative damage and p70S6K signaling pathway in human GSCC. The present study may provide an experimental basis for ZnO-NPs to be considered as a promising novel anti-tumor agent for the treatment of gingival cancer.

## 1. Introduction

Oral squamous cell carcinoma (oral cancer; OSCC) is a malignant neoplasm and its high incidence and mortality rates are particularly relevant in certain parts of Europe and South-Eastern Asia [1]. The character of OSCC is a high degree of local infiltration and it has a high rate of recurrence and metastasis for the cervical lymph nodes [2,3]. The current treatments of OSCC contain surgical resection, radiation therapy, chemotherapy, and targeted therapy. Despite advances in diagnostic technology and treatment strategy, the prognosis of OSCC remains poor, especially in late stage. More than 60% of patients with OSCC have late-stage disease (stage III or IV) resulting from the difficult diagnosis, especially gingival squamous cell carcinomas (GSCC), which pretend as benign disease both clinically and histologically [4,5,6]. Therefore, there is an urgent need to discover effective strategies for the treatment of human GSCC.

Accumulated studies have demonstrated that the production of reactive oxygen species (ROS), including peroxide, superoxide, peroxynitrite, and hydroxyl radicals, is highly associated with apoptosis of cancer cells [7,8]. The induction and/or reduction of ROS generation plays a vital role in cell physiology. High level cellular ROS dramatically promotes oxidative damage to various cells, resulting in apoptosis and cell death [9]. Furthermore, cancer cells are also sensitive to exogenous ROS-generating compounds through the production of intracellular ROS levels [10]. Therefore, more understanding for the impact of ROS will provide promising strategies for the development of novel and efficacious cancer therapies [11].

The emerging field of nanotechnology provides better opportunities for diagnosis, imaging, and cancer treatment. Zinc oxide nanoparticles (ZnO-NPs) are metal nanoparticles that are widely used in commercial products, including dental materials and cosmetic and pharmaceutical products, due to their unique physicochemical properties [12,13,14]. Recently, ZnO-NPs have been shown to serve as potential agents for their broad anti-cancer property against several cancer types, including hepatocellular carcinoma, head and neck squamous cell carcinoma, non-small cell lung cancer, prostate cancer, and colorectal cancer [15,16,17]. The anti-cancer effects of ZnO-NPs result from the inhibition of the proliferation of cancer cells, the increase of the sensitivity for drug-resistant cancer cells, the prevention of tumor invasiveness and metastasis, as well as the restoration of cancer immunosurveillance [18]. However, the anti-cancer effect and mechanism of ZnO-NPs on human GSCC have not been thoroughly investigated to date. The purpose of this study was to determine whether ZnO-NPs exert an anti-cancer property on gingival cancer cells. Furthermore, the role of superoxide, mitochondrial function, and signal pathway in the cytotoxicity of ZnO-NPs for human GSCC was also investigated.

## 2. Results

### 2.1. ZnO-NPs Inhibited Cell Growth of Gingival Squamous Cell Carcinomas

**Figure 1:**
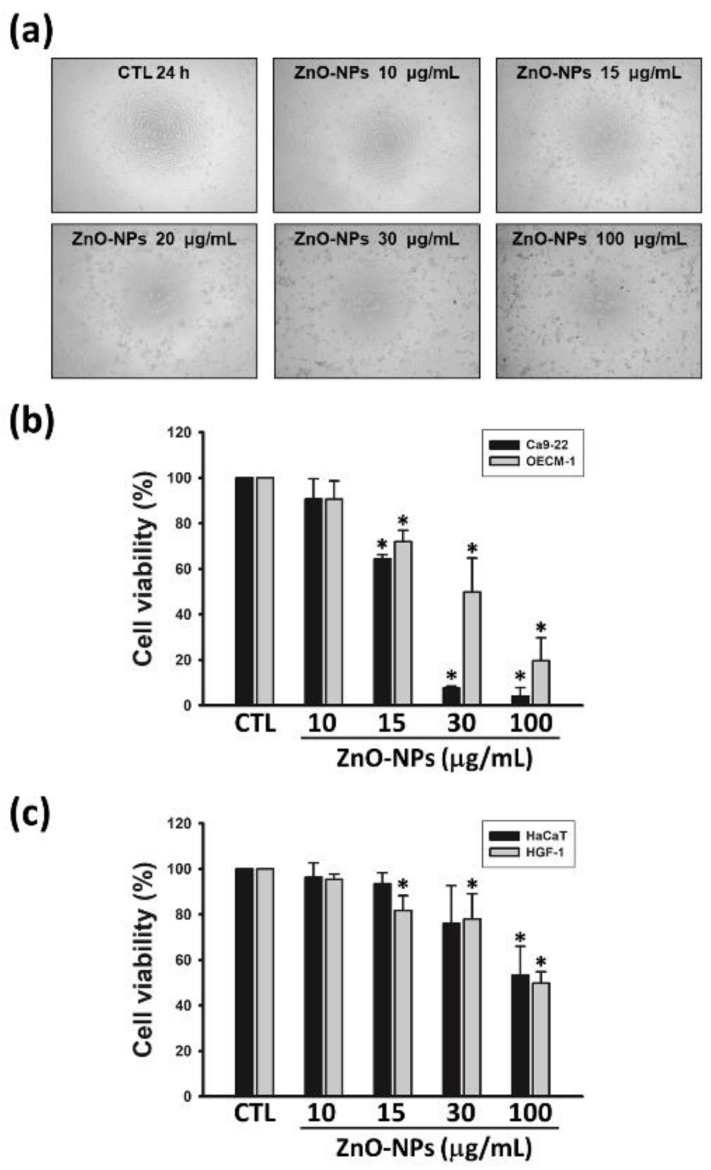
Effects of zinc oxide nanoparticles (ZnO-NPs) on cell growth in human gingival squamous cell carcinoma (GSCC) and normal cells. Ca9-22 or OECM-1 cells were incubated with the indicated concentrations of ZnO-NPs for 24 h. The cell morphology (**a**) and viability (**b**) were assessed by the inverted phase contrast microscope and MTT assay, respectively. (**c**) Human normal keratinocyte cells (HaCaT) and gingival normal cells (HGF-1) were treated with the indicated concentrations of ZnO-NPs for 24 h, then cell viability was determined using MTT assay. Data represent the mean ± SEM of four independent experiments. * *p* < 0.05 compared with the control group.

We first examined the growth-inhibitory effect of ZnO-NPs in human GSCC cell lines (Ca9-22 and OECM-1 cells). The cell morphology changes were visualized under the treatment of various concentrations of ZnO-NPs in Ca9-22 cells (Figure 1a). ZnO-NPs concentration-dependently inhibited cell growth of Ca9-22 and OECM-1 cells (Figure 1b), with IC_50_ values of 17.4 ± 0.6 and 51.0 ± 0.6 μg/mL, respectively. In parallel, ZnO-NPs were less toxic to normal cell types, including human normal keratinocytes (HaCaT cells) and gingival fibroblasts (HGF-1 cells) (Figure 1c). In addition, the growth inhibition of ZnO-NPs in gingival cancer cells was more effective than other types of human cancer cell lines (Figure S1). These results demonstrated that ZnO-NPs induce a selective anti-cancer effect against human GSCC.

### 2.2. ZnO-NPs Caused Sub-G1 Arrest and Apoptosis in Gingival Squamous Cell Carcinomas

**Figure 2:**
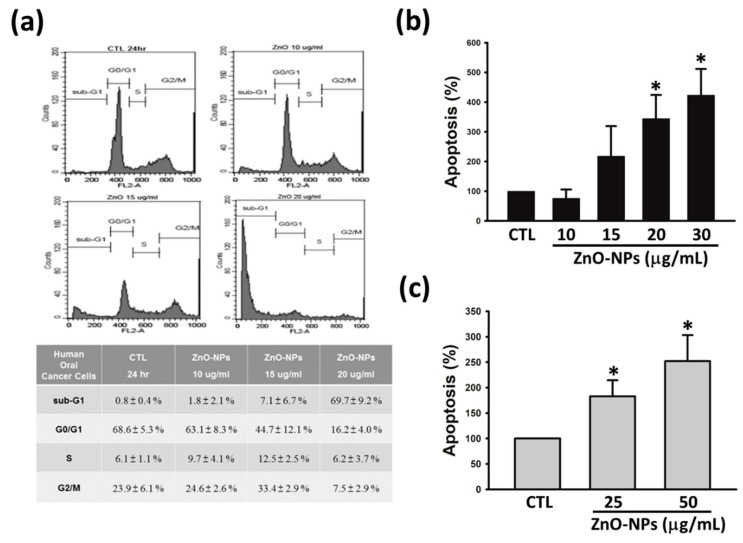
Effects of ZnO-NPs on cell cycle progression and apoptosis in human GSCC cells. (**a**) Ca9-22 cells were treated with ZnO-NPs (10–20 µg/mL) for 24 h, and subsequently analyzed by propidium iodide (PI) staining to determine the cell cycle distribution. Lower panel, quantitative data were based on histograms. Ca9-22 (**b**) and OECM-1 (**c**) cells were treated with the indicated concentrations of ZnO-NPs for 24 h to determine apoptosis using a Cell Death ELISA^PLUS^ kit. Data are expressed as the mean ± SEM of at least three independent experiments. * *p* < 0.05 compared to the control group.

To elucidate the mechanism of growth inhibition on gingival cancer cells, the effects of ZnO-NPs on cell cycle progression were determined in Ca9-22 cells. Figure 2a shows the sub-G1 population percentiles of gingival cancer cells was increased after exposure to ZnO-NPs for 24 h, as compared to the control group. The results of quantitative analysis revealed that ZnO-NPs induced significant concentration-dependent accumulation of cell cycle at sub-G1 phase. Accumulated studies indicate that targeting apoptotic signaling pathway is the crucial therapeutic strategy for cancer treatment [19]. Hence, we next evaluated whether apoptosis was involved in ZnO-NP-mediated anti-cancer effect in human GSCC. As shown in Figure 2a,c, ZnO-NPs exhibited a concentration-dependent induction of histone-associated DNA fragmentation in both Ca9-22 and OECM-1 cells. These results indicated that ZnO-NPs induce sub-G1 arrest of the cell cycle followed by apoptosis in human GSCC.

### 2.3. ZnO-NPs Stimulated ROS and Superoxide Generation in Gingival Squamous Cell Carcinomas

**Figure 3:**
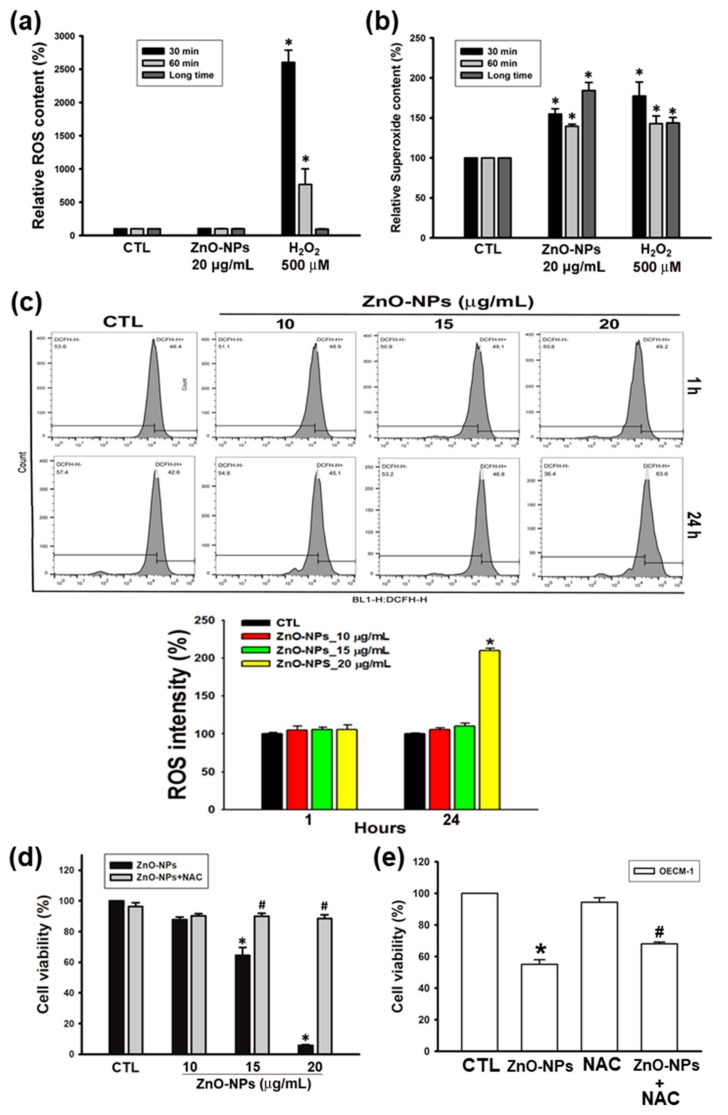
Effects of ZnO-NPs on the production of reactive oxygen species (ROS) and superoxide in human GSCC cells. (**a**,**b**) Ca9-22 cells were incubated with ZnO-NPs for short-term (30–60 min) and long-term (24 h) treatments. Then, the production of ROS and superoxide was examined by ROS/superoxide detection kit. H_2_O_2_ was used as a positive control. (**c**) Ca9-22 cells were treated with ZnO-NPs for the indicated times to determine the intracellular ROS by flow cytometry analysis. Representative flow cytometry-based ROS patterns are shown. The quantification of ROS intensity was analyzed using CellQuest software. (**d**,**e**) Ca9-22 and OECM-1 cells were treated with the indicated concentrations of ZnO-NPs with or without *N*-acetyl-L-cysteine (NAC) (5 mM) for 24 h. Then, the cell viability was determined using MTT assay. Data are expressed as mean ± SEM of four independent experiments. * *p* < 0.05 compared with control group; # *p* < 0.05 compared with ZnO-NP-treated group.

Several studies have shown that the cytotoxicity of ZnO-NPs results from the generation of ROS [20,21,22]. We first used the ROS/superoxide detection kit to determine the impact of ZnO-NPs on oxidative stress in gingival cancer cells. Using two fluorescent probes for the measurement of total levels of ROS and superoxide specifically, we found that ZnO-NPs did not increase the global levels of ROS (Figure 3a), but induced the significant superoxide production at short-term (30–60 min) and long-term (24 h) treatments of ZnO-NPs (Figure 3b). To validate the effects of ZnO-NPs on intracellular ROS, the levels of ROS were analyzed using the DCFH-DA probe by flow cytometry analysis. As shown in Figure 3c, ZnO-NPs profoundly increased the content of ROS in a concentration-dependent manner after 24 h of treatment. Furthermore, our results illustrated that *N*-acetyl-L-cysteine (NAC) significantly prevented ZnO-NP-induced cell death of Ca9-22 and OECM-1 cells (Figure 3c,d). Therefore, these findings demonstrated that ROS production, especially superoxide anion radical, is essential for the anti-cancer activity of ZnO-NPs in human GSCC.

### 2.4. ZnO-NPs Triggered Mitochondrial Intrinsic Apoptosis in Gingival Squamous Cell Carcinomas

**Figure 4:**
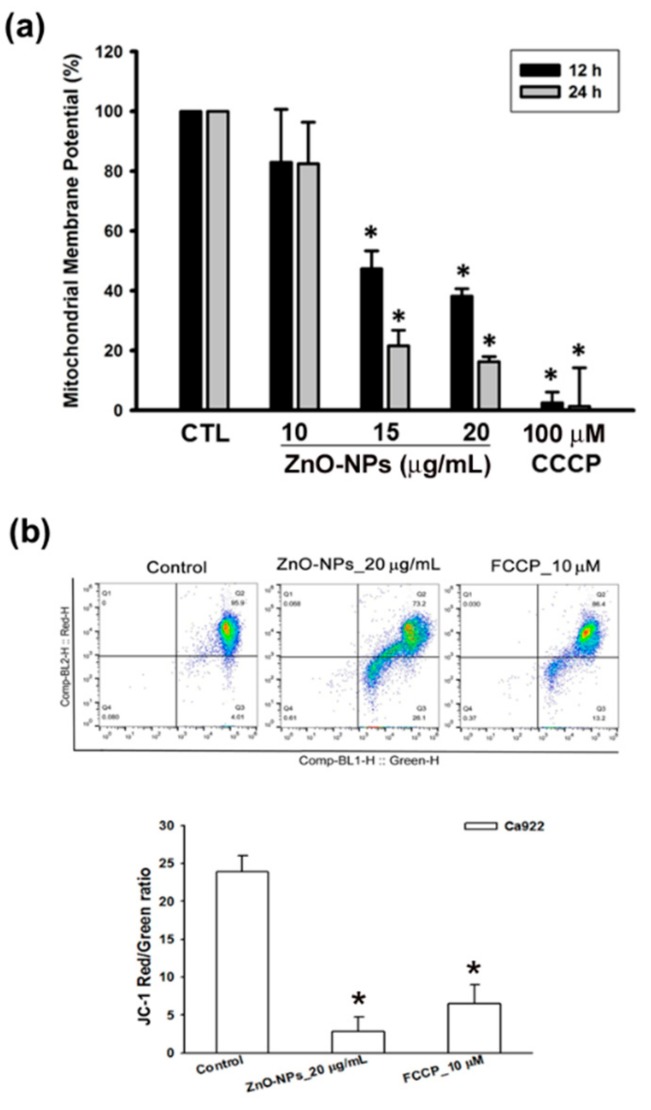
Effects of ZnO-NPs on mitochondrial membrane potential of human GSCC cells. (**a**) Ca9-22 cells were treated with ZnO-NPs (10–20 µg/mL) and carbonyl cyanide m-chlorophenyl hydrazone (CCCP) (100 μM) for the indicated times to determine the change of mitochondrial membrane potential using MITO-ID assay kit. (**b**) Flow cytometry analysis showed the gating of JC-1 (red) aggregates and JC-1 (green) monomer populations in Ca9-22 cells treated with ZnO-NPs and carbonyl cyanide-4-(trifluoromethoxy)phenylhydrazone (FCCP) for 24 h. Ratio of JC-1 staining represents the mitochondrial function. Data are expressed as mean ± SEM of four independent experiments. * *p* < 0.05 compared with control group.

Disruption of mitochondrial membrane potential (MMP) is the hallmark of apoptosis typography. We further determined whether ZnO-NPs triggered apoptotic cell death through the mitochondrial pathway. The data showed that ZnO-NPs induced a significant loss of the MMP in Ca9-22 cells using an MITO-ID assay kit and JC-1 staining (Figure 4). Carbonyl cyanide m-chlorophenyl hydrazone (CCCP) and carbonyl cyanide-4-(trifluoromethoxy)phenylhydrazone (FCCP), mitochondrial oxidative phosphorylation uncouplers, were used as positive controls for the analysis of MMP. Bcl-2 family proteins play an important role in the MMP loss and mitochondria-related apoptosis. In addition, we found that ZnO-NPs did not affect the expression of pro-survival Bcl-2 members (Bcl-2, Bcl-xl, and Mcl-1) as well as pro-apoptosis Bcl-2 members (Bax, Bad, and Bid) in Ca9-22 cells (Figure S2). Taken together, we suggest that mitochondrial dysfunction is involved in ZnO-NP-induced apoptosis in human GSCC. 

### 2.5. ZnO-NPs Induced Caspase-Dependent Apoptosis in Gingival Squamous Cell Carcinomas

**Figure 5:**
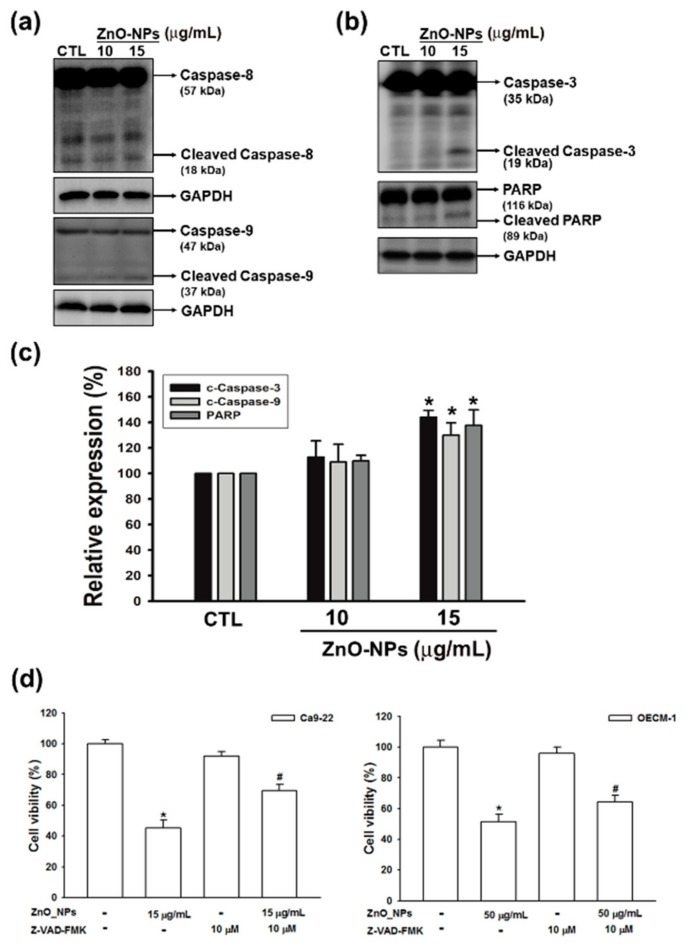
Effects of ZnO-NPs on caspase cascade in human GSCC cells. Ca9-22 cells were treated with the indicated concentrations of ZnO-NPs for 24 h. Then, cells were harvested and lysed for the detection of initiator caspase (caspase -8 and -9) (**a**), caspase-3, and poly-(ADP-ribose) polymerase (PARP) (**b**) by Western blot analysis. (**c**) The quantitative densitometry of cleaved form of the indicated caspases and PARP was performed with Image-Pro Plus. (**d**) Percentage cell viability assessed by MTT assay in GSCC cells, which were treated with ZnO-NPs in the presence or absence of Z-VAD-FAK. Data represent the mean ± SEM of three independent experiments. * *p* < 0.05 compared with the control group, # *p* < 0.05 compared to ZnO-NP-treated cells.

Caspases, a family of cysteine acid proteases, are central regulators for cell survival and apoptosis [23]. To investigate whether ZnO-NP-induced apoptosis is associated with the caspase cascade, we next analyzed the expressions of initiator caspase (caspases-8 and -9) and effector caspase (caspase-3), and its downstream substrate of poly-(ADP-ribose) polymerase (PARP) in ZnO-NP-treated Ca9-22 gingival cancer cells. The results showed that ZnO-NPs did not significantly induce the activation of caspases-8, but the cleavage of caspase-9 was clearly increased after ZnO-NPs treatment (Figure 5a). Meanwhile, the cleavage of caspase-3 and PARP were also profoundly activated by ZnO-NPs (Figure 5b,c). Moreover, the general caspase inhibitor (Z-VAD-FAK) reversed the cytotoxicity of ZnO-NPs in both Ca9-22 and OECM-1 cells (Figure 5d). Our data indicated that ZnO-NPs promoted cell apoptosis through the caspase-dependent pathway. Collectively, we propose that superoxide-induced mitochondrial dysfunction is associated with the ZnO-NP-mediated caspase-dependent apoptosis in human GSCC.

### 2.6. ZnO-NPs Inhibited p70S6K Signaling Pathway in Gingival Squamous Cell Carcinomas

**Figure 6:**
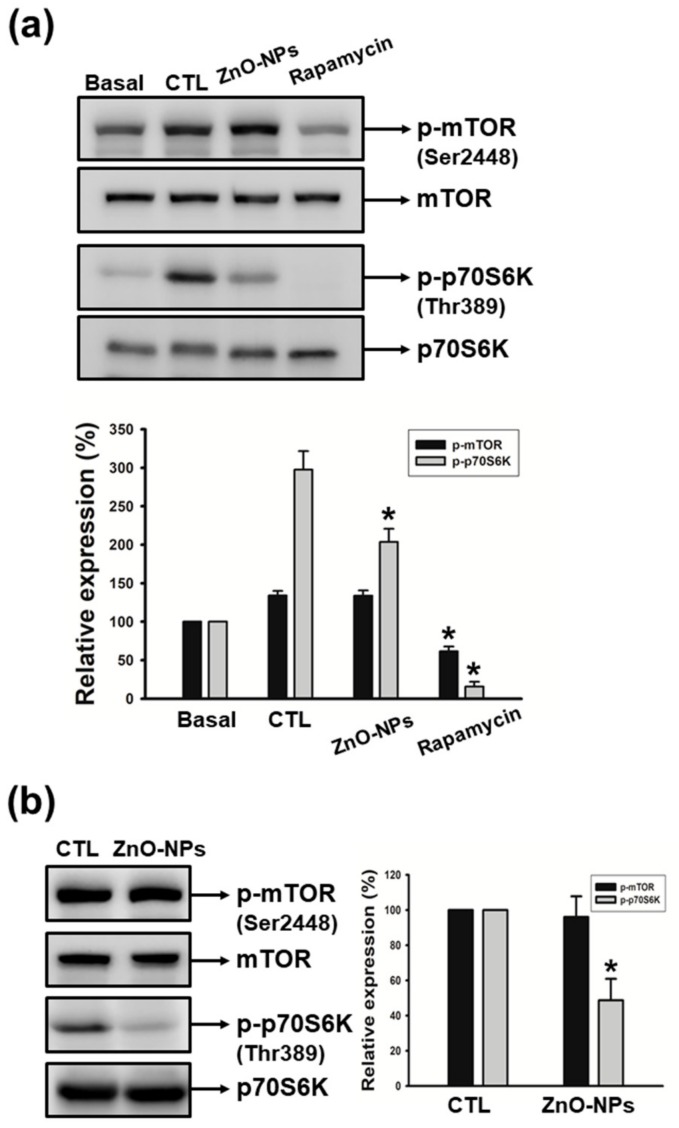
Effects of ZnO-NPs on the activation of mTOR and p70S6K in human GSCC cells. (**a**) Quiescent Ca9-22 cells were treated with or without culture medium (10% fetal bovine serum (FBS)) in the absence (CTL) or presence of ZnO-NPs (20 µg/mL) or rapamycin (10 µM). (**b**) Quiescent OECM-1 cells were treated with ZnO-NPs (50 µg/mL). Then, cells were harvested and lysed for the detection of *p*-mTOR and *p*-p70S6K by Western blot analysis. Image-Pro Plus processing software quantified the relative level of phosphorylated protein. Data represent the mean ± SEM of five independent experiments. * *p* < 0.05 compared with the control group.

The mammalian target of rapamycin (mTOR) signaling pathway is important for cell growth, leading to the facilitation of cancer progression [24,25]. Ribosomal protein S6 kinase (p70S6K) is a well-known modulator for the translational pathway that generally dominates cell death or survival. In order to investigate the mechanisms underlying ZnO-NP-induced apoptosis, we evaluated the impact of ZnO-NPs on mTOR and p70S6K kinases in human GSCCs. As shown in Figure 6a, culture medium-containing serum induced a significant increase in the phosphorylation of mTOR and p70S6K, whereas rapamycin (a specific mTOR inhibitor) dramatically suppressed these serum-induced effects. We found that ZnO-NPs significantly inhibited the phosphorylation of p70S6K, but did not abolish the phosphorylation of mTOR in both Ca9-22 and OECM-1 cells (Figure 6a,b). These findings revealed that ZnO-NPs may induce apoptotic cell death through the p70S6K signaling pathway in human GSCC. The signal of mTOR was found to not be involved in ZnO-NPs’ anti-cancer effect.

### 2.7. ZnO-NPs Impeded Tumor Growth of Gingival Squamous Cell Carcinomas in Vivo

**Figure 7:**
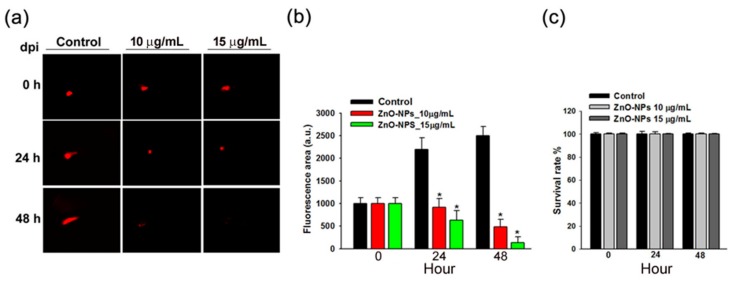
Effects of ZnO-NPs on tumor growth of Ca9-22 cells in zebrafish xenograft model. (**a**) The intensity of red fluorescence is proportional to the xenograft tumor size. *n* = 20 embryos for each group. The scale bar is 16× magnification. (**b**) The quantitative analysis for the anti-tumor efficacy of ZnO-NPs. (**c**) The survival rate of the zebrafish xenograft model after ZnO-NP treatment is shown. Data represent the mean ± SEM of three independent experiments. * *p* < 0.05 compared to the vehicle-treated control group.

We further evaluated the anti-tumor efficacy of ZnO-NPs in vivo. Ca9-22 cells were implanted into the yolk sac of zebrafish larvae followed by incubating with different ZnO-NPs concentrations for the indicated times. We found that the observed tumor sizes, as indicated by the intensity of red fluorescence, were reduced under the treatment of ZnO-NPs (Figure 7a). After quantification, the data showed that ZnO-NPs inhibited tumor growth in zebrafish inoculated with Ca9-22 cells in a dose-dependent manner (Figure 7b), thereby indicting the anti-tumor effects of ZnO-NPs in the zebrafish xenograft model. Moreover, the survival rate of zebrafish embryos was not affected by ZnO-NPs (Figure 7c), suggesting that ZnO-NPs at testing doses did not cause obvious toxicity to the zebrafish embryos.

## 3. Discussion

**Figure 8:**
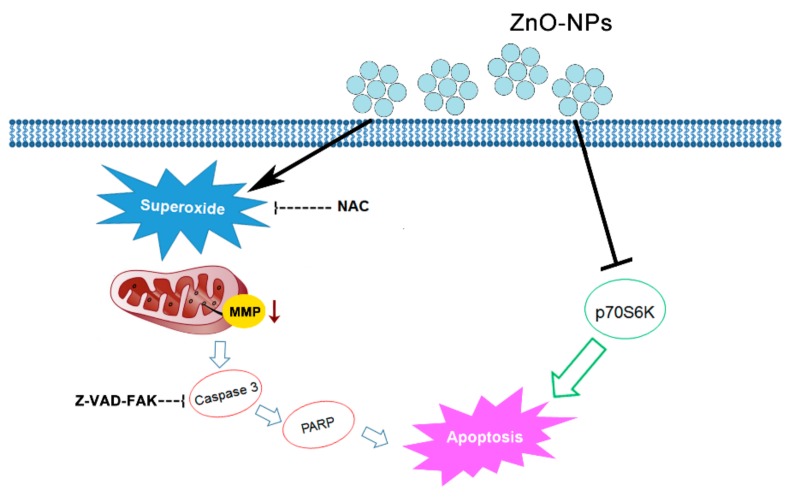
Schematic diagram of ZnO-NP-induced anti-cancer mechanism in human GSCC. Our study data indicate that ZnO-NPs may promote caspase-dependent apoptosis via mechanisms involving both superoxide-mediated mitochondrial intrinsic and p70S6K signaling pathways.

ZnO-NPs are multi-functional metal oxide NPs due to various biological purposes such as anti-bacterial and anti-ultraviolet products [26]. The recent important role of ZnO-NPs in tumor therapy has developed great interest. In the present study, a detailed investigation was conducted on the anti-cancer effects of ZnO-NPs in human GSCC, both in vitro and in vivo. As revealed by MTT assay, the viability of Ca9-22 and OECM-1 gingival cancer cells was concentration-dependently inhibited by ZnO-NPs, but no obvious cytotoxic fashion was found in normal cells. In addition, ZnO-NPs dramatically caused cell cycle arrest at the sub-G1 phase. The production of ROS and specifically superoxide was increased by ZnO-NPs, and the loss of MMP was subsequently observed in gingival cancer cells. Under the quantitative assessment of oligonucleosomal DNA fragmentation, ZnO-NPs were identified as increasing cell apoptosis. Importantly, the phosphorylation of p70S6K was downregulated, and the caspase cascade was activated by ZnO-NPs. On the basis of the findings herein, we propose that ZnO-NPs may promote caspase-dependent apoptosis via the superoxide-induced mitochondrial dysfunction and p70S6K signaling pathway in gingival cancer cells (Figure 8). Notably, ZnO-NPs exerted a promising anti-tumor effect in the zebrafish xenograft model. This is the first demonstration that ZnO-NPs exhibit an anti-cancer property against gingival cancer in vitro and in vivo. ZnO-NPs may serve as a potential therapeutic candidate for the treatment of human oral cancer, especially GSCC.

Previous studies have demonstrated that ZnO-NPs possess anti-tumor activity via induction of ROS and depletion in antioxidant system, which is considered as one of the mechanistic pathways of ZnO-NPs in causing cancer cell death [27,28]. High levels of ROS lead to a reduction in the function of anti-oxidative enzymes in the cells, and eventually results in an oxidative damage to cells and/or tissues [29]. It has been shown that ROS can regulate the translocation, phosphorylation, and/or cleavage of pro-apoptosis Bcl-2 members (Bax, Bak, Bad, Bid, Bim, etc.), leading to the induction of apoptosis [30]. Pro-apoptosis Bcl-2 family proteins also induce the permeabilization of outer mitochondrial membrane, as well as modulate mitochondrial homeostasis for contributing to the loss of MMP [31]. Interactions between death-promoting and death suppressing Bcl-2 family members have led to a rheostat model in which the ratio of pro-apoptotic and anti-apoptotic proteins control cell fate. Additionally, the intrinsic pathway of apoptosis is also initiated by mitochondrial membrane permeabilization, which is activated by various types of oxidative stress, especially superoxide [32]. Several studies have demonstrated that ZnO-NP-induced cytotoxicity in cancer cells is associated with ROS production [22,33]. In this study, our data showed that ZnO-NPs profoundly increased the content of intracellular ROS, especially superoxide anion radical, and subsequently disrupted mitochondrial function in gingival cancer cells. Meanwhile, this study proposes that the role of superoxide is important for ZnO-NP-mediated mitochondrial oxidative damage and apoptosis in human GSCC. Nevertheless, ZnO-NPs did not impair the protein expression of pro-survival Bcl-2 members (Bcl-2, Bcl-xL, and Mcl-1) and pro-apoptosis Bcl-2 members (Bax, Bad, and Bid). These results indicate that ZnO-NP-mediated MMP loss and cell apoptosis are not regulated by altering the balance of pro-survival and pro-apoptosis Bcl-2 members. Several studies have shown that increased Bax/Bcl-2 ratio induces the activation of caspase 3, which in turn increases apoptosis. However, some contradictory reports reveal that Bax translocation without Bax/Bcl-2 ratio alteration can upregulate caspase 3 and induce apoptosis [34,35,36]. Therefore, Bax/Bcl-2 ratio is important in inducing mitochondria-dependent apoptosis; however, it is not absolutely required. Whether ZnO-NPs affect other pro-apoptosis Bcl-2 members (e.g., Bak, Bok, as well as BH3 subfamily Bik, Bim, Noxa, and Puma) with their anti-cancer effect should be further investigated in the future.

Apoptosis has been reported as processing through several mechanisms, including alteration of the intracellular mitochondrial pathway to induce the activation of caspase cascade [37]. The activation of the extrinsic pathway is regulated by caspase-8, which in turn cleaves and activates effector caspases. In parallel, the intrinsic pathway requires disruption of the mitochondrial membrane and the release of mitochondrial proteins including Smac/DIABLO, HtRA2, and cytochrome c. Cytochrome c interacts with Apaf-1 to conduct the activation of caspase-9, thereby initiating the cleavage of downstream caspase-3 and PARP for apoptotic cell death. Here, we showed that ZnO-NPs significantly induced the cleavage of caspase-3, caspase-9, and PARP for executing cell apoptosis. Furthermore, treatment with specific caspase inhibitor obviously prevented ZnO-NP-induced cell death in gingival cancer cells. These results demonstrated that ZnO-NPs activate caspase-9, leading to the stimulation of effector caspase-3, suggesting that a mitochondrial intrinsic apoptosis pathway is involved in ZnO-NP-mediated apoptosis. Additionally, anti-oxidant agent NAC significantly reversed ZnO-NP-induced cytotoxicity. Therefore, we suggest that ZnO-NP-induced apoptosis may be caused by superoxide formation via the mitochondrial intrinsic pathway in gingival cancer cells. 

In mammalian cells, p70S6K plays a key role in translational control of cell proliferation in response to growth factors. The mTOR/p70S6K translational pathway is recognized as being a pivotal modulator of cellular functions [25,38]. Furthermore, p70 S6 kinase is also required for cell growth and cell cycle progression under normal physiologic conditions [39]. On the contrary, the abnormal activation of mTOR and p70S6K kinases has been reported as dictating cancer development in various human tumors [40]. Therefore, targeting of the mTOR/p70S6K pathway is becoming an attractive therapeutic strategy for cancer treatment [41,42]. For example, puerarin inhibits cell proliferation of bladder cancer via the mTOR/p70S6K signaling pathway [43]. Bufalin impedes the p70S6K pathway, and promotes apoptosis in esophageal squamous cell carcinoma [44]. Carboplatin represses the expression of mTOR, and the phosphorylation of its major downstream effector p70S6K, for inducing ovarian cancer cell apoptosis [45]. In addition, the p70S6K-specific inhibitor PF-4708671 exhibits an inhibitory effect on non-small cell lung cancer in both in vitro and in vivo models [46]. Interestingly, ZnO-NPs suppressed the phosphorylation of p70S6K, but had no obvious inhibition on the phosphorylated level of mTOR, which therefore excluded the role of mTOR in ZnO-NP-induced apoptosis on gingival cancer cells. Rapamycin, a well-known mTOR inhibitor, dramatically repressed the phosphorylation of mTOR and p70S6K, and these results are consistent with previous studies on rapamycin. Therefore, we suggest that the anti-cancer mechanism of ZnO-NPs is different from rapamycin, and that p70S6K may be considered a therapeutic target in human GSCC. The mTOR signal is the important upstream effector of p70S6K for translation control and a wide variety of cell functions. However, extracellular signal-related kinase (ERK) and phosphoinositide-dependent protein kinase 1 (PDK1) have been reported as regulating the activity of p70S6K for signal transduction. p70S6K kinase acts downstream of PDK1 to recapitulate PDK1’s action in various biological functions of cells [47]. In addition, p70S6K has been shown to serve as the downstream of ERK in IL-6-induced epithelial to mesenchymal transition (EMT) and metastasis [48]. In the present study, our results demonstrated that mTOR is not involved the cytotoxicity of ZnO-NPs in gingival cancer cells. Whether ZnO-NPs inhibit the activation of p70S6K through ERK and/or PDK1 signaling pathways with their anti-cancer property requires further investigation. 

## 4. Materials and Methods

### 4.1. Particle and Physicochemical Properties

ZnO-NPs were purchased from UniRegion Bio-Tech (New Taipei City, Taiwan), and the morphology of ZnO-NPs was observed under a scanning electron microscope (SEM; Hitachi S-4700) with an accelerating voltage of 15 kV (Figure S3). Particles were coated with a platinum film by sputtering physical vapor deposition. ZnO-NPs were dispersed in dimethyl sulfoxide (DMSO), without any surfactant or modification, for all assays. DMSO was used as the vehicle control in the experiments.

### 4.2. Cell Culture

The human gingival squamous cell carcinoma (GSCC) cell line (Ca9-22) and gingival normal cell line (HGF-1) were obtained from Health Science Research Resources Bank (HSRRB, Osaka, Japan). The human GSCC OECM-1 cell line was purchased from EMD Millipore Corporation (Temecula, CA, USA). The normal human keratinocytes (HaCaT) cell line was provided by Prof. N.L. Wu (MacKay Memorial Hospital, Taipei, Taiwan). Cells were maintained in Dulbecco’s modified Eagle’s medium (DMEM) containing 10% fetal bovine serum (FBS), penicillin (100 units/mL), and streptomycin (100 µg/mL) under humidified air containing 5% CO_2_ at 37 °C. All cell culture reagents were purchased from Gibco-BRL life technologies (Grand Island, NY, USA).

### 4.3. Cell Viability Assay

Cells were plated in 96-well microtiter plates and treated with various concentrations of ZnO-NPs for 24 h, and cell viabilities were assessed using MTT (Sigma-Aldrich, St. Louis, MO, USA) colorimetric assay.

### 4.4. Cell Cycle Analysis

Cells were treated with ZnO-NPs for 24 h. Then, cells were collected and fixed with 70% ethanol. The cell cycle distribution was analyzed by FACScan flow cytometric analysis according to previously described procedures [49].

### 4.5. Measurement of ROS and Superoxide Production

Cells seeded in black 96-well plates at a density of 2 × 10^4^ cells per well. After treatment of ZnO-NPs, the contents of ROS and superoxide were determined in live cells using a ROS/superoxide detection kit (Enzo Life Sciences, Plymouth Meeting, PA, USA) on the basis of the method of our previous work [50].

### 4.6. Measurement of Intracellular ROS Content

The DCFH-DA probe was used to measure the intracellular ROS levels. After treatment of ZnO-NPs, cells were reacted with DCFH-DA (100 nM) for 30 min at 37 °C. After harvesting and washing, cells were resuspended in PBS for ROS detection using FACScan flow cytometric analysis.

### 4.7. Determination of Apoptosis

ZnO-NP-mediated apoptosis was determined using Cell Death ELISA^PLUS^ kit (Roche, Mannheim, Germany). The method was carried out according to an established protocol [49].

### 4.8. Mitochondrial Membrane Potential (MMP) Analysis

Ca9-22 cells seeded in black 96-well plates at a density of 2 × 10^4^ cells per well. The changes of MMP were analyzed by Mito-ID assay kit (Enzo Life Sciences, Plymouth Meeting, PA, USA) according to previously described procedures [50].

### 4.9. Western Blot Analysis

Total protein was determined and equal amounts of protein were separated by 10%–15% SDS-PAGE and immunoblotted with specific primary antibodies. Antibodies specific for phospho-mTOR (Ser2448), mTOR, and GAPDH were purchased from Abcam (Cambridge, MA, USA). Antibodies specific for phospho-p70S6K (Thr389), p70S6K, caspase-3, caspase-8, caspase-9, PARP, Bcl-2, Bcl-xl, Mcl-1, Bax, Bad, and Bid were purchased from Cell Signaling Technologies (Boston, MA, USA). The process of Western blot was performed by a standard method, as described previously [51].

### 4.10. Detection of MMP By JC-1 Staining

Cells were seeded into 6-well plates at a density of 10^5^ cells/well and cultured at 37 °C with 5% CO_2_ for 24 h. Cells were treated ZnO-NPs and FCCP for 24 h at 37 °C and stained with a JC-1 probe (Sigma-Aldrich) for 5 min at 37 °C according to the manufacturer’s instructions. The prepared cells were detected by flow cytometry and analyzed using CellQuest software (BD Biosciences, San Jose, CA, USA).

### 4.11. Zebrafish Xenograft Assay

The zebrafish (*Danio rerio*) *Tg(fli1:EGFP)* were obtained from Taiwan Zebrafish Core Facility at the National Health Research Institute (NHRI, Miaoli, Taiwan). The care and maintenance of zebrafish were handled in compliance with the animal care regulations and standard protocols of the animal center (Kaohsiung Medical University, Kaohsiung, Taiwan) for zebrafish adults and larvae. Zebrafish were kept at 28.5 °C in aquaria with day/night light cycles (10 h dark vs. 14 h light periods). The use of zebrafish complied with the principles of 3Rs (reduction, replacement and refinement), and the approval protocol by the Institutional Animal Care and Use Committee (IACUC) of Kaohsiung Medical University. Ca9-22 cells were labeled with DiI dye (Molecular Probes, Carlsbad, CA, USA) and injected into zebrafish in order to track the cells using fluorescence microscopy. The procedure was performed according to a previous study [8].

### 4.12. Statistical Analyses

Data are presented as the mean ± SEM for the indicated number of separate experiment. The significance of difference between the experimental groups and controls was assessed by Student’s *t*-test. The difference was considered significant if the *p*-value was < 0.05.

## Supplementary Materials

The following are available online at https://www.mdpi.com/1422-0067/21/5/1612/s1: Figure S1: Effects of ZnO-NPs on cell growth in human cancer cell line panel screening. Figure S2: Effects of ZnO-NPs on Bcl-2 family proteins in Ca9-22 cells. Figure S3: SEM images of ZnO particles.

## Abbreviations

ZnO-NPs	Zinc oxide nanoparticles
GSCC	Gingival squamous cell carcinoma
CCCP	Carbonyl cyanide m-chlorophenyl hydrazone
FCCP	Carbonyl cyanide-4-(trifluoromethoxy)phenylhydrazone
NAC	N-acetyl-L-cysteine
MMP	Mitochondrial membrane potential
PARP	Poly-(ADP-ribose) polymerase
