# Supplementary material for: ZnO Nanoparticles Induced Caspase-Dependent Apoptosis in Gingival Squamous Cell Carcinoma through Mitochondrial Dysfunction and p70S6K Signaling Pathway
Source: Int J Mol Sci. 2020 Feb 26;21(5):1612. doi: 10.3390/ijms21051612 (PMC7084801; doi:10.3390/ijms21051612)
Supplement: Supplementary file 1 [file ijms-21-01612-s001.pdf]

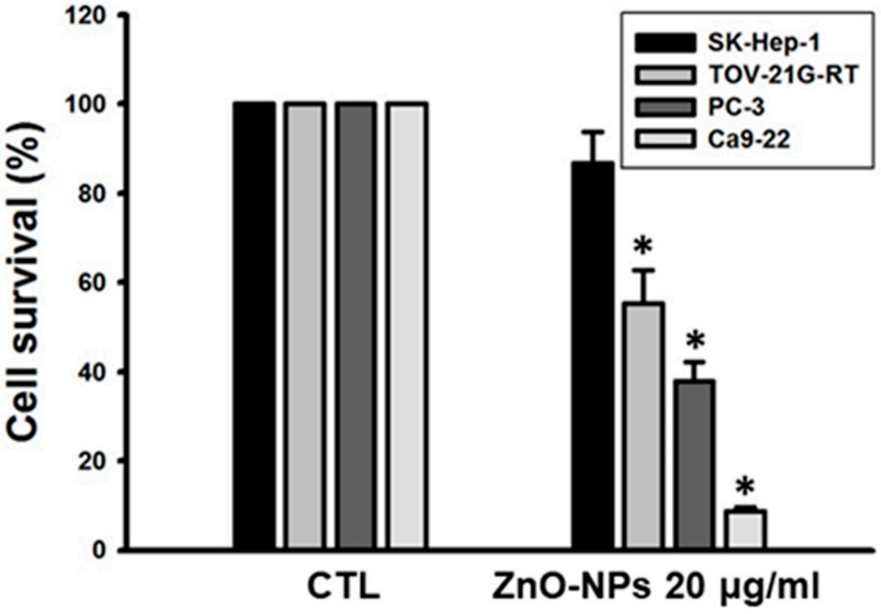

Suppl. Figure 1. Effects of ZnO-NPs on cell growth in human cancer cell line panel screening.

The human oral squamous cancer Ca9-22 cells, prostate cancer PC-3 cells, hepatocellular carcinoma SK-Hep-1 cells, and paclitaxel-resistant ovarian cancer TOV-21G (TOV-21G-RT) cells were treated with ZnO-NPs for 24 h. then cell viability was analyzed by MTT assay. Data represent the mean ± S.E.M. of 4 independent experiments. *$p < 0.05$ compared with the control group.

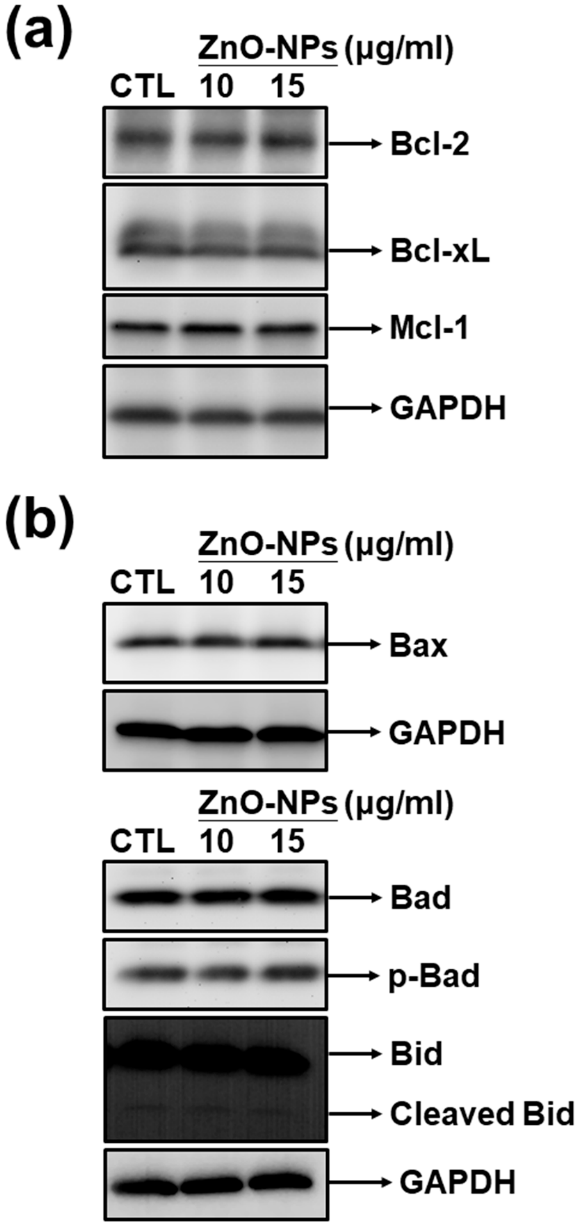

Suppl. Figure 2. Effects of ZnO-NPs on Bcl-2 family proteins in Ca9-22 cells. Cells were treated with the indicated concentrations of ZnO-NPs for 24 h. Then, anti-apoptotic Bcl-2 members (a) and pro-apoptotic Bcl-2 members (b) were determined by Western blot analysis. Blots were representative of results from six independent experiments.

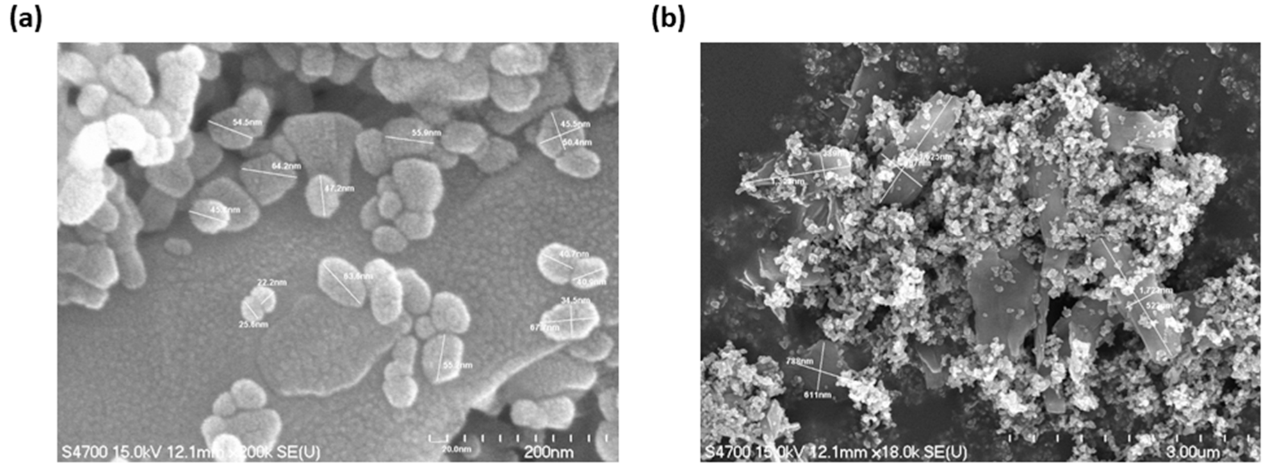

Suppl. Figure 3. SEM images of ZnO particles. The morphology of ZnO-NPs was examined under a SEM. In our measurement system, two groups of ZnO particles with different sizes were observed. (a) The mean diameter of major group of ZnO-NPs is about 55 nm (N=92), whereas (b) the mean diameter of secondary group is about 975 nm (N=11), which could be aggregated due to air dry of DMSO in the preparation for SEM.
